# Clinician perspectives on the multilevel impacts of Pediatric early warning systems (PEWS) in resource-variable hospitals

**DOI:** 10.3389/fonc.2025.1573360

**Published:** 2025-06-17

**Authors:** Amela Siječić, Alejandra Catalina Quesada-Stoner, Sayeda Islam, Sara Malone, Maria F. Puerto-Torres, Adolfo Cardenas Aguirre, Kim Prewitt, Maria do Céu Diniz Borborema, Andreia Ribeiro Pereira Aguiar de Paula, Laura Lemos de Mendonça e Fontes, Silvio Fabio Torres, Leticia Aradi Andrade Sarmiento, Ever Fing Soto, Rosdali Diaz-Coronado, Yefry A. Aragón-Joya, Jose Miguel Mijares Tobias, Isidoro Ejocote Romero, Norma Lopez-Facundo, Scheybi Miralda-Méndez, María Sánchez-Martín, Daniela Arce Cabrera, Daniela María Velásquez Cabrera, Veronica Soto Chávez, Valentine Jimenez Antolinez, Erika Montalvo, Bobbi J. Carothers, Dylan Graetz, Carlos Acuña, Douglas A. Luke, Virginia McKay, Asya Agulnik, The INSPIRE Study Group

**Affiliations:** ^1^ Department of Global Pediatric Medicine, St. Jude Children’s Research Hospital, Memphis, TN, United States; ^2^ Brown School, Washington University in St. Louis, Saint Louis, MO, United States; ^3^ Division of Public Health Sciences, Department of Surgery, Washington University in St. Louis School of Medicine, St. Louis, MO, United States; ^4^ Department of Pediatric Intensive Care, Instituto de Medicina Integral Prof. Fernando Figueira, Recife, Brazil; ^5^ Department of Pediatric Intensive Care, Hospital de Amor, Hospital Infanto Juvenil de Cancer de Barretos, Barretos, Brazil; ^6^ Pediatric Oncology, Hospital Martagão Gesteira, Salvador, Brazil; ^7^ Department of Pediatric Intensive Care, Hospital Universitario Austral, Pilar, Argentina; ^8^ Department of Pediatric Oncology, Unidad de Investigación Médica en Epidemiología Clínica (UMAE) Hospital de Pediatría CMN Siglo XXI, Mexico City, Mexico; ^9^ Department of Pediatric Oncology, Hospital General Celaya, Celaya, Mexico; ^10^ Instituto Nacional de Enfermedades Neoplasicas, Lima, Peru; ^11^ Department of Pediatrics, Instituto Nacional de Cancerología, Bogota, Colombia; ^12^ Department of Pediatric Intensive Care, Hospital de Especialidades del Niño y La Mujer “Dr. Felipe Nuñez Lara”, Queretaro, Mexico; ^13^ Department of Pediatric Oncology, ISSEMYM Materno Infantil, Toluca, Mexico; ^14^ Department of Pediatric Intensive Care, Hospital Escuela Universitario, Tegucigalpa, Honduras; ^15^ Department of Pediatric Intensive Care, Hospital La Paz, Madrid, Spain; ^16^ Department of Pediatric Oncology, Hospital Pediátrico de Sinaloa, Culiacán, Mexico; ^17^ Department of Pediatrics, Hospital Regional De Alta Especialidad Del Bajio, León, Mexico; ^18^ Department of Pediatric Oncology, Hospital Civil de Guadalajara “Dr. Juan I.Menchaca”, Guadalajara, Mexico; ^19^ Department of Pediatric Hematology, Hospital Universitario Dr. José Eleuterio González, Universidad Autonoma de Nuevo León, Monterrey, Mexico; ^20^ Department of Pediatric Intensive Care, Sociedad de Lucha Contra el Cancer (SOLCA) Quito, Quito, Ecuador; ^21^ Associated Department of Pediatrics and Pediatric Surgery, Oriente University of Chile, Providencia, Chile

**Keywords:** pews, resource variable, impact, Latin America, pediatric oncology, Spain, clinician perception, multilevel impact

## Abstract

**Background:**

Pediatric Early Warning Systems (PEWS) are evidence-based interventions that monitor hospitalized pediatric patients to improve outcomes and prevent complications, particularly in children with cancer. However, there is limited data on how clinicians perceive the impact of PEWS on patient care across healthcare centers in resource-variable settings. Understanding clinicians’ perceptions of PEWS is crucial, as their recognition of its benefits can enhance adoption and sustainability across various healthcare settings.

**Objective:**

To assess clinician perceptions of impacts following PEWS implementation across pediatric oncology centers in Latin America and Spain.

**Methods:**

We conducted a secondary analysis of a study assessing capacity for PEWS sustainability and adaptations at resource-variable hospitals participating in a collaborative to implement PEWS. Anonymous surveys in Spanish and Portuguese were distributed to nurses, physicians, ward, and ICU clinicians using PEWS at 58 hospitals across 19 countries. The survey included one free-text question about adaptations made to PEWS. A qualitative analysis of these responses was conducted using codes developed during a previous study to describe clinician perceptions on PEWS impact. Content analysis focused on clinician perspectives on the multilevel impact of PEWS.

**Results:**

Of 1,909 free-text responses, PEWS impact was mentioned in 48% (n=913) by clinicians at 58 participating hospitals. Participants described impacts at the level of the patient, clinician, team, and institution, and emphasized the positive impact of PEWS at their centers. PEWS was perceived as vital in facilitating timely patient care interventions, mitigating progression of critical illness, and reducing mortality for pediatric oncology patients. Clinicians also reported that PEWS made patient care easier and empowered them in their roles. Finally, PEWS was perceived to improve communication and team dynamics among multidisciplinary clinicians.

**Conclusion:**

This study adds to existing literature by describing clinician perceptions of the multilevel impacts of PEWS on hospital care for children with cancer across hospitals of diverse resource-levels, providing further evidence of how this intervention might benefit patients, clinicians, and clinical teams. These findings emphasize that understanding perspectives of clinicians who use evidence-based interventions, like PEWS is crucial to promote adoption and guide sustainability strategies to improve outcomes for children with cancer globally.

## Background

Pediatric oncology patients face many unique and complex healthcare needs that make them vulnerable to life-threatening complications and increase their chances for clinical deterioration during treatment (1, 2). Pediatric Early Warning Systems (PEWS) are evidence-based interventions (EBIs) to monitor and assess hospitalized children’s vital signs and clinical parameters. PEWS uses a scoring tool that activates an action algorithm for clinicians’ intervention when a child’s health is at risk. These EBIs aid in alerting clinicians to pediatric oncology patients at risk of clinical deterioration by regularly assessing their physiological parameters, thus reducing complications and preventing further deterioration (3).

PEWS are highly beneficial for pediatric oncology patients because they enable the early identification and prevention of clinical deterioration, which is especially crucial for children with cancer who are at high risk for rapid health declines. These benefits in pediatric oncology patients have been assessed and validated across multiple settings of care (4–6). However, implementing PEWS in low-resource settings presents challenges and opportunities (7). There is robust multicenter data for mortality reduction following PEWS implementation at variably resourced pediatric oncology centers in Latin America (2). Prior work has also highlighted the impacts of PEWS beyond improving patient survival, including improving patient-clinician and interdisciplinary communication, enhancing clinician confidence, and reducing healthcare costs (3, 8–10). The multilevel impacts of PEWS have been explored from the perspectives of implementation leaders and hospital administration (11). In their study, Mirochnik et al. examine the multilevel impacts of PEWS across five resource-limited pediatric oncology hospitals, focusing on how PEWS affects patient outcomes, clinical practices, team communication, and institutions overall. They find that PEWS benefited patient, clinician, team, and institutional settings. The study also introduces the “PEWS Cycle of Reinforcement,” a framework that demonstrates how positive outcomes at each level of impact reinforce and sustain the ongoing use of PEWS within hospitals (11).

While this work provides useful information on the broad impact of PEWS, there is still limited data on clinicians’ perspectives on its adoption and integration into routine clinical practice within resource-variable settings. Clinicians’ perceptions of PEWS are essential, as they are the ones who adapt their daily practice to effectively integrate the system. Their insights into its effectiveness, challenges, and impact are essential for understanding how well PEWS works and ensuring its continued success in clinical settings. Understanding these perceptions is vital for overcoming challenges in resource-constrained environments, thereby supporting consistent application, and sustaining high standards of patient care. This work underscores the critical role of clinician experience with PEWS in ensuring its ongoing use and sustainability, particularly in resource-limited centers (12, 13). Additionally, previous literature is limited to specific hospital contexts using PEWS; lack of diverse hospital types and country income levels included in prior work restricts applicability of results to other settings (11). Long-term benefits of PEWS depend on continuous PEWS use in patient care; a practice driven mainly by bedside clinicians (2). To facilitate the global scale-up of this effective intervention, it is essential to understand clinician perspectives on how PEWS impacts patient care across diverse clinical settings and hospital types.

This study addresses limitations of prior work by evaluating clinician perceptions of multilevel impact of PEWS on patients, clinicians, and teams across 58 centers with varying resources to inform strategies for scale-up and long-term sustainability in childhood cancer care globally.

## Methods

This study is a secondary analysis of qualitative data from a mixed-methods study investigating adaptations made to PEWS during the phases of planning, piloting, implementation, and sustainability (14). The St. Jude institutional review board approved this study (IRB approval number: 22-0975). We used the consolidated Criteria for Reporting of Qualitative Research (COREQ) guidelines to comprehensively report qualitative methods and findings (15).

### Hospital and participant selection

We previously reported data collection methods, which are briefly described below (13, 16). Participating centers were involved in Project Escala de Valoración de Alerta Temprana (EVAT), a quality improvement collaborative to improve the quality of care for children with cancer who experience critical illness (17, 18). Implementation team leaders from each site participating in Project EVAT were briefed on this study and identified staff to participate. Eligible staff included clinicians using PEWS and the associated action algorithm (**Supplementary 1**) within pediatric oncology. This included nurses and physicians from disciplines such as pediatric oncology and intensive care. Members of the PEWS implementation team who directly supported its use in pediatric oncology units were also included. Additionally, staff involved in the clinical implementation of PEWS who did not provide direct bedside care—such as healthcare administration, data managers, and other clinical staff, like technicians—were eligible to participate, if involved in PEWS implementation or use. These individuals were contacted via email to complete an anonymous electronic survey describing PEWS use in their center. The survey was open for three to four weeks, with regular reminders sent to participants. We did not provide individual incentives.

### Data collection

The survey included a free-text question regarding PEWS adaptations, “In a few sentences, please tell us how EVAT has been adapted or changed in your hospital during the last six months.” The survey was administrated via Qualtrics in Spanish and Portuguese and took about 15 minutes to complete (19). Data collection spanned from June 15, 2021, to March 26, 2023, with surveys completed by participants during distinct phases of PEWS implementation and sustainment. In the parent study, there were a total of 2094 responses, of which 1909 (91%) had free text responses. The average response rate per center was 68.2%, with a range of 8.8% to 100%.

### Analysis

Qualitative analysis of the free-text responses referencing perceived impacts of PEWS was guided by the framework proposed by Mirochnick et al. (11). The study by Mirochnik et al. evaluated staff perceptions regarding the multilevel impact of PEWS at the level of patients, clinicians, teams, and institutions and described how these perceived impacts reinforced ongoing PEWS use. The qualitative codes for this study were based on those used in Mirochnick et al. and modified through iterative analysis of free-text responses to capture responses that described perceived impacts. By refining these codes through an iterative process, we were able to adapt them to our context, ensuring a standardized approach to representing clinician perceptions. The “impacts” code was categorized into six levels: 1) Patients, 2) Clinicians, 3) Team, 4) Institution, 5) Implementation, and 6) Other (See appendix 1 for codebook).

Two multilingual authors (AQS, AS) independently coded individual Spanish and Portuguese responses using the MAXQDA software, achieving a kappa of 0.9 to 0.99 (20). A third author (AA) resolved discrepancies to ensure coding consistency and rigor. Free-text responses were coded and analyzed in the original language (Spanish or Portuguese) but translated to English by the multilingual authors (AQS, AS, AA) for this report. Two multilingual authors (AS, AQS) conducted a content analysis to explore themes related to clinician perceptions of the impacts of PEWS. Although the free-text question did *not* explicitly inquire about PEWS impact, many respondents highlighted their perspectives on various impacts of PEWS at their center.

To further understand the applicability of PEWS, we conducted a frequency analysis and calculated the percentage of respondents who mentioned the impact of PEWS in free-text responses. We assessed these responses across profession, hospital type, and country income-level. Specifically, the number of responses that referenced the impact of PEWS were assessed for each group (e.g., Nurses, Physicians, Public, Private, and mixed hospitals, LMICs, UMICs, and HICs), and the percentage was derived by dividing the number of responses mentioning impacts by the total number of respondents within each category. This approach helped assess variation in perceptions across professions, healthcare settings, and country contexts, offering a clearer understanding of how PEWS is viewed broadly and within specific groups.

## Results

**Figure 1:**
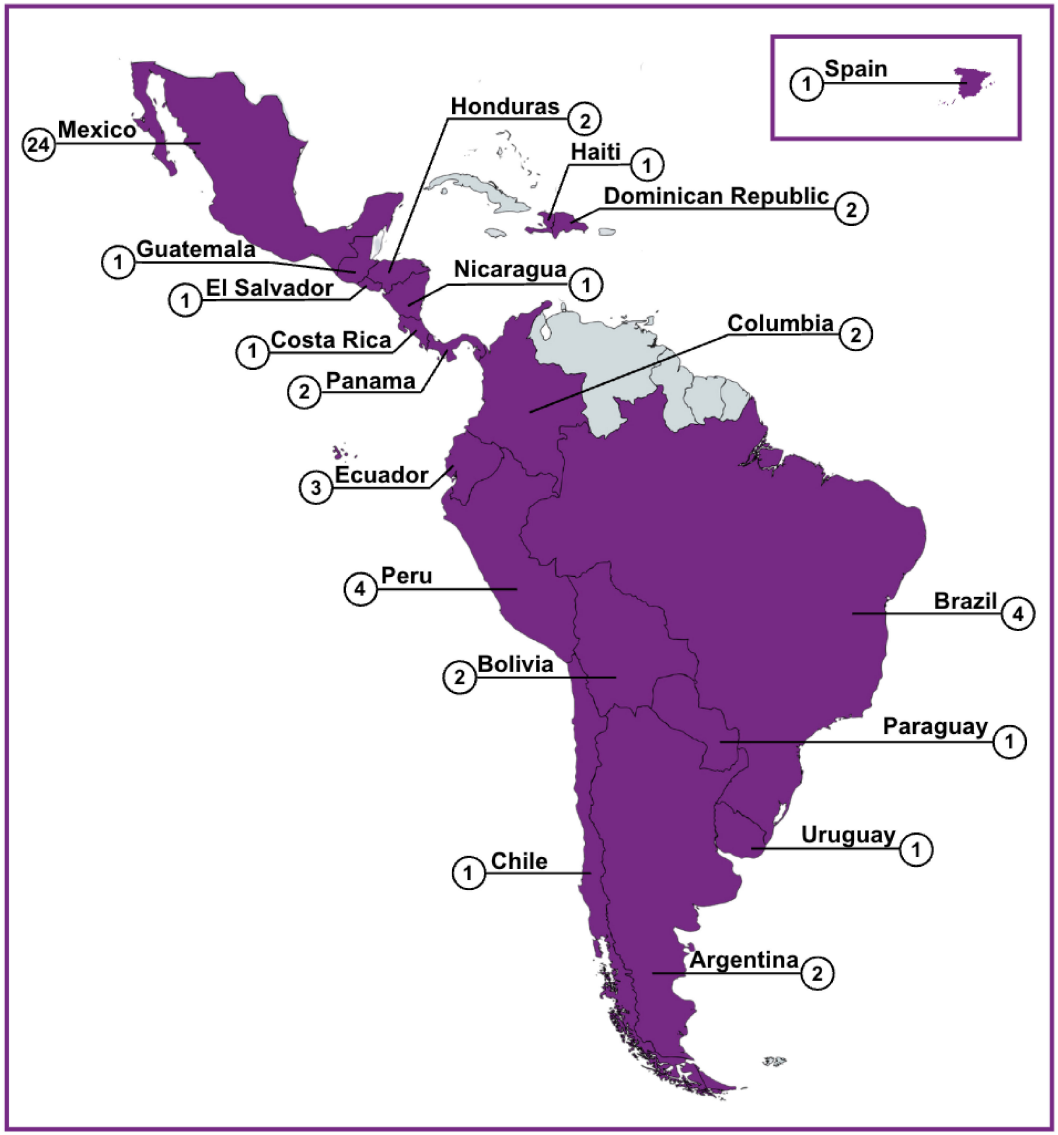
Participating study centers. A map depicting the centers *(n=58)* that participated in the parent study and also had responses that contributed to the secondary analysis.

During the study period, the survey received 1,909 free-text responses from clinicians using PEWS at 58 Project EVAT centers across 19 countries in Latin America and Europe (**Figure 1**). Individual responses varied in length, from a brief statement to 2–4 detailed comments. Of these responses, 913 (48%) mentioned the perceived impacts of PEWS use and were included in this analysis. **Table 1** outlines the participant and center characteristics.

**Table 1:** Participant and center characteristics (of those describing PEWS impact, n=913).

Participant Characteristics	n	%	Center Characteristics	n	%
Profession			Country Income Level		
Nurse	632	69.2	Lower Middle Income	6	10.3
Physician	210	23.0	Upper Middle Income	47	81.0
Other clinical staff*	45	4.9	High Income	3	5.2
Healthcare Administration	23	2.5	Hospital Type		
Data manager/Research	3	0.5	General	21	36.2
Primary Area of Work			Oncology (adult and pediatric)	9	15.5
Pediatric/Oncology Floor	760	83.2	Women and Children’s	4	6.9
Intensive Care Unit	66	7.3	Pediatric Oncology	1	1.7
Other	52	5.7	Funding Type		
Non-Clinical Work	26	2.8	Public	39	67.2
Emergency Department	9	1.0	Private	5	8.6
Role in PEWS			Mixed (Public and Private)	6	10.3
Clinical Staff	656	71.9	Teaching Hospital		
PEWS Leader	164	18.0	Yes	47	81.0
Other	33	3.6	No	3	5.2
Data Managers	33	3.6	Timepoints per center		
Hospital Administration	27	2.9	One timepoint	7	12.1
Length of Work at Hospital (years)			Two timepoints	45	77.6
1-5	320	35.0	Three timepoints	6	10.3
6-10	240	26.4	Observation per timepoint (mean, range)	140	12-245
11-15	128	14.0	Annual New Diagnoses (mean, range)	119	5-800
20<	104	11.4	Nurse-to-Patient ratio (mean, range)	1:6	1:3 -1:12
16-20	85	9.3	Months since PEWS implementation	32	1-96
<1	36	3.9	Completion (mean, range)		
Gender					
Female	790	86.5			
Male	123	13.5			
**Total**	**913**	**100**	**Total (n, %)**	**58**	**100**

*Other clinical staff involved in patient care who are not captured by the other specified categories (e.g., nurse technicians).

The perceived impacts of PEWS were mentioned with similar frequencies by clinicians practicing at different hospital types (private, public, and mixed) and countries with varying resource levels While both nurses and physicians described perceived impacts of PEWS, this was more commonly mentioned in free-text responses by nurse respondents (60% vs. 30%), as shown in **Table 2**. There were no perceived negative outcomes of PEWS mentioned in free-text responses.

**Table 2:** Responses describing pews impact by profession, hospital type, and country income level (n=1901 total free-text responses).

Clinician Type	N free-text responses*	N responses mentioning PEWS impact	% responses mentioning PEWS impact
Profession			
Nurse	1,056	632	59.8%
Physician	691	210	30.1%
Other staff	162	71	43.8%
Hospital Type			
Private	251	109	43.4%
Public	1292	648	50.2%
Mixed**	366	156	42.6%
Country Income Level			
LMIC	92	55	59.8%
UMIC	1658	784	47.3%
HIC	159	74	46.5%

*These are the total responses from the parent study (16).

**Includes hospitals that are a combination of private and public funding.

**Figure 2:**
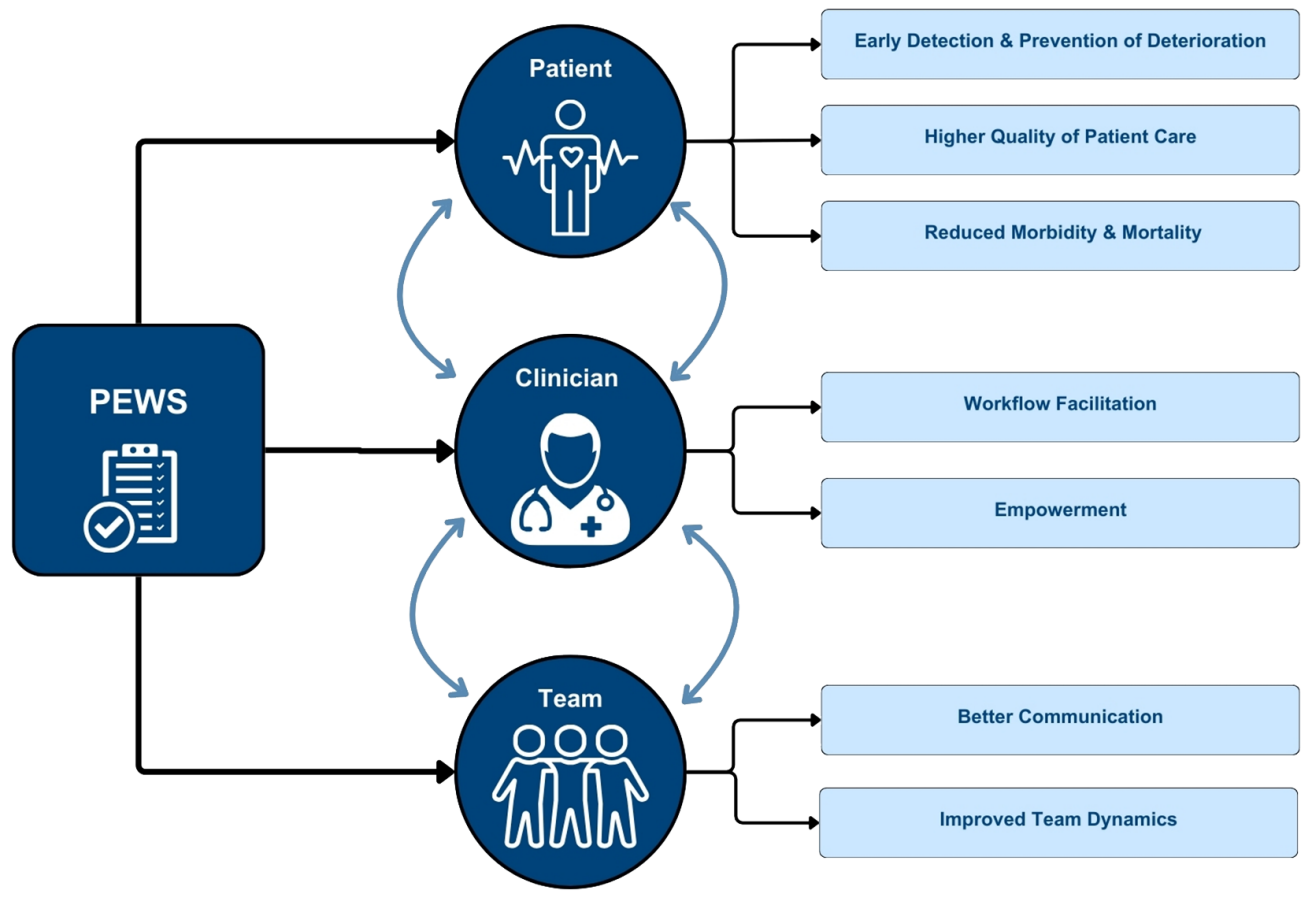
Multilevel Impact of PEWS. This figure describes identified themes related to clinician perceptions of the multi-level impact of PEWS on patients, clinicians, and teams. Additionally, each of these levels reinforce the positive impacts of PEWS at the other levels.

Qualitative analysis identified themes regarding the perceived benefits of PEWS for patients, clinicians, and teams. **Figure 2** depicts the modified framework proposed by Mirochnik, et al. identifying multilevel impacts associated with PEWS, further explained below. All identified themes were similar across respondents of different professions, hospital types, and country income-level.

### Impact on patients

Participants mentioned numerous benefits from PEWS at the *patient* level, including early detection and prevention of deterioration, overall higher quality of patient care, and reduction in mortality (**Table 3**).

**Table 3:** Patient-level impacts.

Theme	Participant quote
Early Detection & Prevention of Deterioration	“It [PEWS] has allowed early interventions in cancer patients to avoid serious outcomes or complications.” (Physician, Ecuador)
	“It [PEWS] has been used to detect complications early in order to make timely interventions.” (Nurse, Panama)
Higher Quality of Patient Care	“It improved the quality of patient care because of the way PEWS requires us to evaluate and monitor a patient.” (Nurse, Mexico)
	“We have achieved better care for our patients.” (Physician, Argentina)
Reduction in Mortality	“It has been adapted in a satisfactory manner, changing and reducing our mortality rate which was 100%.” (Nurse, Peru)
	“Lower mortality, huge changes thanks to PEWS.” (Physician, Bolivia

Earlier detection of deterioration was felt to result partly due to more frequent vital sign assessments. The algorithm used with PEWS requires a vital sign assessment at least every 8 hours. Through facilitating rapid decision-making, clinicians perceived that PEWS allowed them to detect and prevent deterioration effectively, “It helped with more rapid detection of whether a patient had to be brought down to the PICU or treated rapidly within the department” (Nurse, Ecuador). In turn, this prevented further clinical complications, “We have been able to identify patients at high risk of complications earlier, preventing them from more serious effects” (Physician, Mexico).

Clinicians also reported that the increased frequency of vital sign assessments enhanced the quality of care, “The frequency of vital sign assessment has increased, which represents improvements in the quality of care and identification of warning signs” (Physician, Dominican Republic). Clinicians reported that the quality of care also improved as PEWS helped them better recognize and address severe patient complication, “It helped to understand and standardize monitoring for patients prone to complications, helping prevent the collapse or further complication of the patient’s condition” (Nurse, Mexico). Overall, clinicians viewed PEWS as effective for enhancing the quality of patient healthcare, “PEWS is the tool that guides priority and effective actions that healthcare personnel must carry out to ensure quality care and patient welfare” (Nurse, El Salvador).

Participants also mentioned that prior to PEWS, the severity of patient’s symptoms would not be recognized early enough, at times leading to death. More frequent assessment of vital signs led to early identification of deterioration and timely interventions, which reduced mortality from critical illness and improved patient outcomes, “Previously, patients deteriorated significantly and even died. Today, patients no longer have complications; they are addressed and referred to the PICU for better follow-up” (Nurse, Mexico). Multiple respondents noted a visible reduction in mortality after PEWS implementation, “Its implementation [PEWS] has been very beneficial in the hematology-oncology ward since it has reduced the morbidity and mortality of our kids” (Physician, Honduras).

### Impact on clinicians

In addition to the impact on patients, *clinicians* reported that PEWS facilitated workflow and empowered them in their daily clinical responsibilities (**Table 4**).

**Table 4:** Clinician-level impacts.

Theme	Participant quote
Workflow Facilitation	“PEWS has been very helpful in our daily work as it allows us to observe things that we did not before.” (Nurse, Mexico)
	“It [PEWS] helps facilitate work when patients require more care, especially in intensive care treatment.” (Nurse, Ecuador)
Empowerment	“It [PEWS] is a part of the daily routine, and it empowers the nurses and doctors.” (Physician, Panama)
	“It [PEWS] has permitted me to reliably evaluate my patients within and outside of my institution.” (Nurse, Mexico)

Although some participants mentioned initial difficulty adjusting to PEWS, they eventually became a part of their daily workflow, “It was difficult to acclimate to it [PEWS] at the beginning, but little by little they [staff] became familiar with it, now, the whole team uses it, now we all evaluate [patients] with PEWS” (Nurse, Peru) and “The nursing staff adopted it as a routine in their daily work” (Physician, Bolivia). Subsequently, this was perceived to ease clinical work for clinicians, “It has made it easier for us to prevent neurological deterioration and to evaluate children with cancer” (Nurse, Mexico), and “PEWS benefits us since it is easier to detect signs and alarms that the patient may present. Thus, we can take the necessary measures, and above all, provide better quality care” (Nurse, Guatemala). The facilitation of workflow was particularly noted as beneficial by the nursing respondents.

As clinicians recognized the PEW’s utility and observed positive changes in patient impacts, they felt a growing sense of empowerment in their clinical roles. This empowerment was evident across various facets, including enhancement in their overall skillset, “PEWS has provided the staff who provide care with competencies and skills for professional performance” (Nurse, Mexico). Clinicians highlighted that this improvement in their skillset led to a boost in self-confidence, “PEWS has brought great changes in the way nurses and doctors approach patients, as well as empowering nurses through the training they have received” (Nurse, Costa Rica) and “it gives staff a sense of security when evaluating patients” (Other clinical staff, Honduras). The use of PEWS not only improved patient impacts but also empowered clinicians, enhancing their skills, confidence, and sense of security in patient care.

### Impact on clinical teams

While individual clinicians found PEWS beneficial to their work, it also impacted how clinicians worked together in *teams*. Participants said PEWS use resulted in better interdisciplinary communication and improved teamwork (**Table 5**).

**Table 5:** Team-level impact.

Theme	Participant quote
Better Communication	“Monitoring has been much better in all patients, keeping us within the green light [PEWS algorithm] and improving communication between doctors and nurses.” (Nurse, Mexico)
	“It (PEWS) has permitted better communication between the doctors and nurses.” (Physician, Nicaragua)
Improved Teamwork	“In the multidisciplinary team, we accept it as a program that has brought many benefits for the organization and multidisciplinary teamwork… (Nurse, Mexico)
	“Better coordination, good multidisciplinary team coordination.” (Nurse, Peru)

PEWS facilitates clear and organized communication between multidisciplinary clinicians when evaluating patients. This collaborative work was perceived to bring positive impacts for teams with better communication between the ward and intensive care unit when caring for at-risk patients, “Adapting [to PEWS] during the last year has been excellent, with better communication with the nursing unit and the pediatric intensive care unit” (Physician, Mexico), and between nurses and physicians; “PEWS has progressively brought changes for the better, improving communication between the multidisciplinary team” (Nurse, Ecuador). Clinicians observed that PEWS enhanced both daily operations and collaborative dynamics among healthcare providers.

This not only improved communication between units but also facilitated a structured approach to teamwork, “Effective communication, increasing teamwork” (Nurse, Peru). The use of PEWS in hospitals improved the structure and operations of clinical work, improving team collaboration, “The implementation of PEWS in our institution was extremely significant, bringing order, more systematized teamwork…” (Nurse, Argentina). One clinician described how PEWS impacted teamwork by identifying that it aided their daily activities, leading to increased confidence, “Alerts were solved in a timely manner, increasing confidence in our work, and improving teamwork” (Nurse, Peru).

### Institutional impact

In contrast to patient, clinician, and team benefits, clinicians rarely mentioned additional PEWS benefits at the level of the institution. One participant discussed institutional cost savings because of PEWS. Routine PEWS use was felt to reduce resources needed for patient care, thus decreasing overall institutional costs, “Since [PEWS] was implemented a few years ago … it reduced the use of certain resources and is cost-effective” (Other clinical staff, Haiti).

## Discussion

This study explores clinician perspectives on the impacts of PEWS in the care of hospitalized pediatric oncology patients across diverse resource-variable settings. The perception of providers align with results of previous research showing both the perceived impacts and demonstrated benefits of PEWS in early detection and prevention of patient deterioration, enhancing care quality, reducing morbidity and mortality, streamlining workflow, empowering clinicians, and fostering improved communication and teamwork (2, 8–12). Our study provides additional insights to how these perceived clinician impacts are generalizable across resource-variable settings, promoting the adoption, sustainability, and global scale-up of PEWS.

This analysis builds upon the framework proposed by Mirochnik et al. based on a study of five centers across four countries, focusing primarily on the perspectives of PEWS implementation leaders and hospital directors. However, the limited scope of this study raised questions about its broader applicability (11). Our study expanded on this work by applying the framework to free-text survey responses by clinicians using PEWS in patient care. Instead of responding to direct questions about PEWS benefits, clinicians spontaneously reported the perceived benefits highlighted in this study. Our study captured perspectives from clinicians at 58 diverse centers worldwide, spanning lower-middle-income to higher-income countries and including both public and private hospitals. Similar to Mirochnik et al., our study demonstrates that PEWS provides multilevel benefits for patients, clinicians, and healthcare teams. However, in contrast to Mirochnik et al., our study results did not identify strong perceived institutional benefits such as cost savings, awards, and collaboration opportunities. However, this outcome aligns with our focus on clinicians, who prioritize their personal experiences with PEWS rather than institutional advantages. Clinicians naturally emphasized their day-to-day interactions with PEWS when not prompted to consider broader organizational impacts. By examining a broader, more diverse population of hospitals and their clinicians, our study confirmed the relevance of Mirochnik et al.’s framework in describing the multilevel impact of PEWS across different settings. Our findings reinforce the framework’s applicability across diverse healthcare environments and highlight its effectiveness in capturing consistent patterns in clinician-reported benefits.

The survey providing data for this study was designed to examine how hospitals adapted PEWS to ensure long-term use, encouraging clinicians to reflect on PEWS sustainability (16). Since clinicians were not directly asked about the impacts of PEWS, they naturally focused on their personal experiences, which led to the spontaneous identification of its perceived benefits. Previous research on pediatric early warning systems has shown that sustainability depends largely on clinician buy-in, with staff more motivated to use them if they view them as clinically relevant and meaningful in practice (21, 22). In prior work, our team has also highlighted that staff resistance poses a significant barrier to successful and sustained implementation of interventions like PEWS, underscoring the need for strategies to address this challenge (7, 23). Given that clinician buy-in is crucial for PEWS maintenance, clinician perceptions that PEWS eases their workload and improves care practices promotes PEWS continued use, reinforcing the importance of fostering positive perceptions of effective interventions for sustainability (12, 14).

Our study reinforces that clinicians perceive PEWS as a valuable EBI, particularly within pediatric oncology care, where early detection and timely intervention are crucial for improving patient outcomes. These findings align with existing literature and offer practical insights for hospitals and researchers focused on intervention sustainability. By leveraging our results, clinicians, implementors, and researchers can develop targeted strategies to strengthen clinician engagement, facilitating the transition from PEWS adoption to long-term integration into routine patient care. Understanding factors that drive clinician buy-in enables healthcare organizations to ensure PEWS sustainability and maximize its impact on patient care. Additionally, the broad applicability of these results suggests that the benefits of PEWS can extend across a wide range of healthcare settings, regardless of resource availability or hospital type.

Our study has several limitations. As a secondary analysis of a study focused on adaptations made to PEWS, the study’s primary aim was not to evaluate clinician perceptions of PEWS impacts. However, the frequent mention of these impacts by respondents emerged as an inductive theme during the qualitative analysis, prompting this investigation. A key limitation of this study is that we did not explicitly ask participants about the impacts of PEWS, which may have led to an underestimation of perceived benefits. Some participants may not have mentioned their views or may have provided limited detail without direct prompting. However, because these impacts were reported spontaneously, the findings are less likely to be influenced by social desirability bias, which may offer a more authentic reflection of participants’ experience. Additionally, the cross-sectional design of this study limited participants to providing insights from a single point in time, preventing examination of how these perceived impacts evolved or were sustained over time. Finally, the use of free-text responses introduces interpretive limitations, as respondents cannot elaborate on their answers, which may result in an incomplete or skewed understanding of their intended meaning.

In conclusion, this study highlights clinician perceptions of the multilevel impacts of PEWS in pediatric oncology, emphasizing its benefits for patients, clinicians, and clinical teams. The consistency of responses across clinician groups, hospital types, and varying levels of income underscores the universal applicability of PEWS in improving patient outcomes, enhancing clinical practice, and fostering team collaboration. By focusing on insights from bedside clinicians, these findings provide valuable guidance for developing strategies to sustain and expand PEWS globally to reduce disparities in childhood cancer outcomes. Future research should investigate how these perceived impacts evolve over time and evaluate their role in supporting long-term PEWS sustainability.

## Data Availability

The raw data supporting the conclusions of this article will be made available by the authors, without undue reservation.
