# Supplementary material for: Clinician perspectives on the multilevel impacts of Pediatric early warning systems (PEWS) in resource-variable hospitals
Source: Front Oncol. 2025 Jun 17;15:1573360. doi: 10.3389/fonc.2025.1573360 (PMC12209193; doi:10.3389/fonc.2025.1573360)
Supplement: Supplementary file 1 [file DataSheet1.pdf]

Pediatric Early Warning System Scoring Tool

St. Jude Children's Research Hospital
ALSAC • Danny Thomas, Founder
Finding cures. Saving children.

PASHA Scoring tool

	0	1	2	3	Result
**Neurologic / Behavioral**	° Alert / Sleeping Appropiatley<br>° Patient is at baseline consciousness / awareness state	° Sleepy / drowsy if unstimulated<br>° Responds to Verbal Stimuli	° Irritable, unconsolable.<br>° Responds to painful stimuli.	° Lethargic / confused<br>° Unresponsive<br>° New, frequent or prolonged seizure<br>° Anisocoria or unreactive.	
**Cardiovascular**	° Appropiate skin color<br>° Capillary refill <3s.<br>° Normal pulses.	° Pale.<br>° Vasodilated*<br>° Capilary refill 3-4s<br>° Mild Tachycardia	° Capilary refill 5s.<br>° Moderate Tachycardia.<br>° Diminished periferal pulses	° Mottled<br>° Capilary refill >5s<br>° Severe Tachycardia<br>° Symptomatic Bradicadrdia<br>° Arrythmia	
**Respiratory**	° Normal or baseline Work of Breathing (WOB) pattern.<br>° No retraccions / respiratory distress<br>° Spo2 >93% or within baseline.	° Mild Tachypnea*<br>° Mild respiratory distress (Nasal flaring and /or intercostal retractions only)<br>° Up to 1 LPM oxygen use (Nasal prongs)<br>° SpO2 90%-93% or within 5% lower than baseline;	° Moderate Tachypnea<br>° Moderate respiratory disress (previous signs plus accespry muscle use and / or grunting)<br>° Oxygen use >1 and up to 3 LPM or Use of simple facemask.<br>° Short acting, nebulized medication use every 4hrs or longer.<br>° SpO2 88-89% without oxygen or within 10% lower than baseline.	° Severe Tachypnea*<br>° Bradipnea*<br>° Severe respiratory distress (Any of previous signs plus Head-Bobbing, toraco-abdominal dissociation)<br>° Oxygen use > 3LPM or use of Non rebreather mask.<br>° Short acting, nebulized medication use more frequently than every 4hrs.<br>° SpO2 < 90% WITH oxygen deliver or more tan 15% under baseline.<br>° Apnea	
**Nurse / Staff Concern**	Unconcerned	Concerned			
**Family / concern**	Unconcerned AND present	Concerned OR Absent.			
				**Total Result**	

*** For Tachypnea and Tachycardia, refer to the Vital Signs Reference Tool.**
Based on Bonafide C, et al. Development of Heart and Respiratory Rate Percentile Curves for Hosptalized Children. Pediatrics 2013;131;e1150.

	MILD	MODERATE	SEVERE
**Respiratory and / or Heart Rate.**	90-95% pc for age	95-99% pc for age	> 99% pc for age
