# Supplementary material for: Clinician perspectives on the multilevel impacts of Pediatric early warning systems (PEWS) in resource-variable hospitals
Source: Front Oncol. 2025 Jun 17;15:1573360. doi: 10.3389/fonc.2025.1573360 (PMC12209193; doi:10.3389/fonc.2025.1573360)
Supplement: Supplementary file 2 [file DataSheet2.pdf]

Pediatric Early Warning System Evaluation Algorithm

St. Jude Children's Research Hospital
ALSAC • Danny Thomas, Founder
Finding cures. Saving children.

0-2
(Green)

- **Continue routine asesment.**

3-4
(Yellow)

- **Monitor VS and PEWS q ____ hours**
- **Notify Nurse supervisor and attending physician.**
- **Asses patient evolution multidisciplinarily.**
- **Consider referal to step up unit.**
- **Document interventions**

Additional Criteria to consider as if Yellow

- **Need for 2 or more fluid boluses in the last 4 hours.**
- **Uncontrolled pain or pain inconmensurate with clinical condition.**

Treatment determined by hospitalist.

Consider PICU or equivalent consultation

5 or >
(Red)

- **Monitor VS and PEWS q ____ hours**
- **Bedside Physician assesment.**
- **Notify Nurse supervisor and attending physician.**
- **Notify treating physician**
- **Asses patient evolution multidisciplinarily**
- **Document interventions**

Additional criterio to consider as if Red.

- **Patient requires constant nursing care for > 1 hour**
- **Hypotension.**

Mandatory PICU (or equivalent) consultation.

For Immediate assistance:

Call Extension ##### / Activate Rapid response team.
